# Evaluation of immunogenicity and cross-reactive responses of  vaccines prepared from two chimeric serotype O foot-and-mouth disease viruses in pigs and cattle

**DOI:** 10.1186/s13567-022-01072-7

**Published:** 2022-07-08

**Authors:** Pinghua Li, Shulun Huang, Jingjing Zha, Pun Sun, Dong Li, Huifang Bao, Yimei Cao, Xingwen Bai, Yuanfang Fu, Xueqing Ma, Kun Li, Hong Yuan, Jing Zhang, Zhixun Zhao, Jian Wang, Keqiang Zhang, Yingli Chen, Qiang Zhang, Shuyun Qi, Zaixin Liu, Zengjun Lu

**Affiliations:** State Key Laboratory of Veterinary Etiological Biology, OIE/National Foot-and-Mouth Disease Reference Laboratory of China, Lanzhou Veterinary Research Institute, Chinese Academy of Agricultural Sciences, Lanzhou, China

**Keywords:** Immunogenicity, cross-reactive responses, chimeric FMDV, vaccines

## Abstract

Foot-and-mouth disease (FMD) remains a very serious barrier to agricultural development and the international trade of animals and animal products. Recently, serotype O has been the most prevalent FMDV serotype in China, and it has evolved into four different lineages: O/SEA/Mya-98, O/ME-SA/PanAsia, O/ME-SA/Ind-2001 and O/Cathay. PanAsia-2, belonging to the O/ME-SA topotype, is prevalent in neighbouring countries and poses the risk of cross-border spread in China. This study aimed to develop a promising vaccine candidate strain that can not only provide the best protection against all serotype O FMDVs circulating in China but also be used as an emergency vaccine for the prevention and control of transboundary incursion of PanAsia-2. Here, two chimeric FMDVs (rHN/TURVP1 and rHN/NXVP1) featuring substitution of VP1 genes of the O/TUR/5/2009 vaccine strain (PanAsia-2) and O/NXYCh/CHA/2018 epidemic strain (Mya98) were constructed and evaluated. The biological properties of the two chimeric FMDVs were similar to those of the wild-type (wt) virus despite slight differences in plaque sizes observed in BHK-21 cells. The structural protein-specific antibody titres induced by the rHN/TURVP1 and wt virus vaccines in pigs and cows were higher than those induced by the rHN/NXVP1 vaccine at 28–56 dpv. The vaccines prepared from the two chimeric viruses and wt virus all induced the production of protective cross-neutralizing antibodies against the viruses of the Mya-98, PanAsia and Ind-2001 lineages in pigs and cattle at 28 dpv; however, only the animals vaccinated with the rHN/TURVP1 vaccine produced a protective immune response to the field isolate of the Cathay lineage at 28 dpv, whereas the animals receiving the wt virus and the rHN/NXVP1 vaccines did not, although the wt virus and O/GXCX/CHA/2018 both belong to the Cathay topotype. This study will provide very useful information to help develop a potential vaccine candidate for the prevention and control of serotype O FMD in China.

## Introduction

Foot-and-mouth disease (FMD) is a highly contagious, acute vesicular disease that predominantly affects wildlife and domestic cloven-hoofed animals, including cattle, sheep, pigs, goats, and water buffalo [1, 2]. The disease is currently endemic in many parts of the world and often causes devastating economic losses in endemic countries because of the drastic reduction in productivity in adult animals, high mortality among young stock and serious restrictions on the international trade of livestock and animal products [3]. Therefore, FMD still requires global control and eradication [4].

FMD control is largely based on vaccination with an inactivated whole-virus vaccine in endemic areas [4]. The current vaccine has been used for decades and successfully aided in the control of clinical disease in endemic regions and the eradication of FMD from Europe and South America [5, 6]. However, infection and vaccination with one serotype of FMDV does not provide protection against other serotypes and may fail to completely protect against other subtypes within the same serotype [7–9]. Additionally, the emergence of immunologically distinct variants often renders the existing vaccines ineffective owing to failure to provide adequate protection against circulating field strains [10]. Therefore, there is an increasing need to periodically select a new vaccine strain for effective control and eradication of FMD in endemic regions.

Foot-and-mouth disease virus (FMDV), a member of the genus *Aphthovirus* in the family *Picornaviridae*, is the aetiological agent of FMD. The viral genome is composed of a single-stranded positive-sense RNA molecule approximately 8.5 kb in length [11] and contains a 5′ untranslated region (5′ UTR), a single long open reading frame (ORF), a 3′ untranslated region (3′ UTR) and a poly (A) tail. The ORF encodes a single polyprotein, which is processed by three viral proteases (L, 2A and 3C) into seven nonstructural proteins (L, 2A, 2B, 2C, 3A, 3B, 3C and 3D) and four structural proteins (VP4, VP2, VP3 and VP1) [2]. The icosahedral viral capsid is composed of 60 copies of each of the four structural proteins, VP1–VP4. The outer capsid proteins (VP1, VP2 and VP3) of type O FMDV contain five identified neutralizing antigenic sites that are necessary for inducing a complete immunologic response to either vaccination or infection [12, 13]. Among these capsid proteins, VP1 is not only the most genetically hypervariable protein [14] but also a major immunogenic protein [15], which may be attributed to the fact that it contains three of the five antigenic sites. One of them, the highly flexible G-H loop spanning residues 130–160 [16], is an immunodominant site that can induce the production of a high-level neutralizing antibody response in infected and immunized animals, which is important for protection against FMDV infection [17]. Therefore, many studies on FMDV vaccines have mainly focused on the VP1 protein [18–21], and some of them have been proven to be potential candidate vaccines for the prevention and control of FMD.

A previous report revealed that the FMDV O/TUR/5/2009 strain (O/PanAsia-2) is a good match with many FMDVs circulating worldwide, including the newly emerging viruses of the O/Ind-2001 lineage [22, 23]. A recent study also showed that a vaccine prepared from a recombinant FMDV containing the P1 capsid protein O/PAK/44/2008 (PanAsia-2) could provide effective protection against FMDVs of the ME-SA topotype and induce a protective immune response against viruses of the SEA topotype [24]. To develop a better FMDV vaccine that not only protects against all serotype O FMDVs circulating in China but also can be used as an emergency vaccine for the prevention and control of transboundary incursions of PanAsia-2, here we report the development of chemically inactivated FMDV vaccines prepared from two chimeric FMDVs expressing the VP1 capsid protein of the O/TUR/5/2009 strain and the current epidemic strain O/NXYCh/CHA/2018 (Mya-98). The immunogenicity and cross-reactive response of these vaccines against the current circulating serotype O FMDVs of four lineages were evaluated in pigs and cattle.

## Materials and methods

### Cell lines, plasmids and viruses

Baby hamster kidney (BHK-21) cells maintained in minimum essential medium (MEM) (Invitrogen, Carlsbad, USA) supplemented with 10% foetal bovine serum (FBS) (Life Technologies, New York, USA), 1% antibiotics (100 units/mL of penicillin, 20 µg/mL of streptomycin) and nonessential amino acids were used for virus propagation, titration, in vitro growth, plaque assays and neutralization tests. BSR/T7 cells expressing T7 RNA polymerase were propagated in Glasgow minimal essential medium (GMEM) (Life Technologies) containing 10% FBS and 1 mg/mL G418 (Invitrogen) every second passage and were used to recover infectious FMDVs from full-length plasmids. pOFS, a plasmid encoding the complete genome of the FMD vaccine strain O/HN/CHA/93, was used as the genetic backbone for the generation of the recombinants [25], and rHN recovered from pOFS was used as the wild-type (wt) virus [25]. O/Tibet/99 (GenBank AJ539138) belonging to the PanAsia lineage of the ME-SA topotype, O/XJ/CHA/2017 (GenBank MF461724.1) belonging to the Ind-2001 lineage of the ME-SA topotype, O/NXYCh/CHA/2018 (GenBank MH791315.1) belonging to the Mya-98 lineage of the SEA topotype and O/GXCX/CHA/2018 (GenBank MH791316.1) belonging to the Cathay topotype were collected from the regions where FMD outbreaks occurred in China.

### Construction and rescue of the chimeric FMDVs

**Figure 1:**
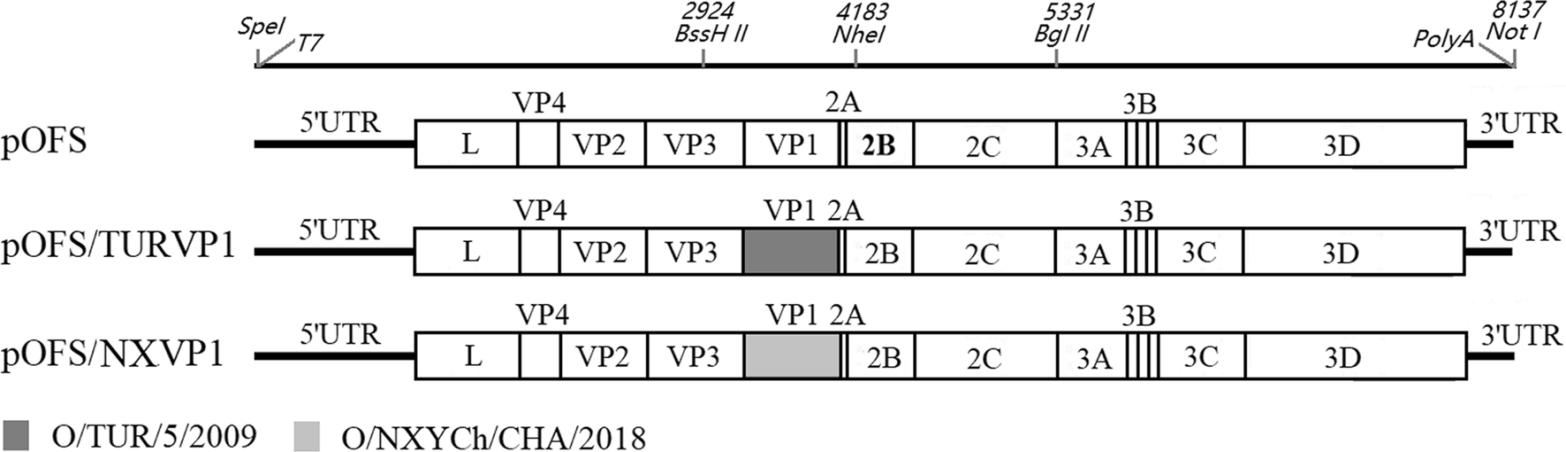
**Schematic diagram of the exchange-cassette cloning strategy described in this study.** The FMDV full-length cDNA clone pOFS was used for the construction of two chimeric genome-length cDNA copies. The specific genes (from nt 2924–4183) with the substitution of the VP1 gene of FMDV O/TUR/5/2009 and O/NXYCh/CHA/2018 were synthesized by a biotechnology company.

The specific gene (nt 2924–4183) of O/HN/CHA/93 containing the VP1 of O/TUR/5/2009 or O/NXYCh/CHA/2018 (Figure 1) was synthesized by a biotechnology company (GENEWIZ, NanJing, China) and cloned into the plasmid pSK-Z123 [25], which was digested with BssH*II* (TaKaRa, DaLian, China) and Nhe*I* (TaKaRa) to generate two recombinant plasmids, pSK-Z123/TURVP1 and pSK-Z123/NXVP1. Then, these plasmids were digested with Spe*I*/Bgl*II* (TaKaRa) and cloned into the corresponding region of pOFS to produce two resulting plasmids, pOFS/TURVP1 and pOFS/NXVP1. The resulting plasmids were sequenced to confirm the presence of expected substitutions. The two recombinant full-length plasmids were linearized with Not*I* (TaKaRa) and purified with a miniBEST DNA fragment purification kit (TaKaRa). The purified samples were transfected into 80–90% confluent BSR/T7 cells using Lipofectamine™ 2000 (Invitrogen) according to the manufacturer’s instructions. At 5 h post-transfection (hpt), 1 mL of complete medium was added, and the transfected cells were further incubated at 37 °C with 5% CO_2_. The cells were examined daily for the appearance of cytopathic effects (CPEs). At 72 hpt, the cell culture supernatants were harvested and then passaged six times in BHK-21 cells. The rescued viruses named rHN/TURVP1 and rHN/NXVP1 were confirmed by sequence analysis and were used for subsequent experiments.

### Morphology of the recombinant FMDVs

To determine the morphology of FMDVs, the mutant viruses and the wt virus (the fifth passage) were cultured in adherent BHK-21 cells. When 100% CPE occurred, the viruses were harvested and centrifuged to remove cell debris. Then, the viruses were inactivated with 5 mM binary ethyleneimine (BEI) for 28 h at 30 °C. The inactivated antigens were clarified by centrifugation and concentrated with 8% polyethylene glycol (PEG) 6000 (Solarbio, Beijing, China) overnight at 4 °C. The pellets were resuspended in TNE buffer (50 mM Tris [pH 7.4], 10 mM EDTA, 150 mM NaCl) and again clarified by centrifugation to remove insoluble particulates. The resulting supernatants were overlaid onto a 10–30% sucrose gradient and then fractionated by centrifugation at 104 000 × *g* for 3 h at 4 °C. The samples were observed by negative-stain electron microscopy as previously described [26].

### Plaque assays and one-step growth kinetics

Plaque morphology of the mutant FMDVs was evaluated in BHK-21 cells. Briefly, confluent BHK-21 cell monolayers seeded in 6-well plates were infected with 200 µL of tenfold serial dilutions of the recombinant viruses or the wt virus. After 1 h of adsorption at 37 °C, the infected cells were washed with PBS (pH = 7.6), overlaid with 2 × MEM supplemented with 2% FBS and 0.6% gum tragacanth, and then incubated at 37 °C for 48 h. The cells were fixed with 50% acetone and 50% methyl alcohol and stained with 1% crystal violet. Plaques were counted, and virus titres were determined.

To determine the possible effect of the substitution of the VP1 gene on virus replication, BHK-21 monolayers were infected with the wt virus or the mutant viruses with a multiplicity of infection (MOI) of 5 and incubated at 37 °C. After 1 h of incubation, the inoculums were removed, and the cells were rinsed three times with PBS, followed by the addition of MEM containing 1% antibiotics (penicillin and streptomycin). At 4, 8, 12, 16 and 20 hpi, the viruses were harvested, and the virus titres were detected by plaque assays in BHK-21 cells using the Reed–Muench method. Titres were determined in duplicate.

### Pathogenesis of FMDVs in suckling mice

Two-day-old BALB/c suckling mice were used to detect the pathogenesis of the mutant FMDVs. The mutant viruses and the wt virus were serially diluted 10 times with MEM. A total of 90 suckling mice were divided into three groups (*n* = 30/group), and every five suckling mice in each group were intraperitoneally inoculated with the same diluted virus (200 µL/mouse). All suckling mice were observed for 7 days after inoculation. The 50% lethal dose (LD50) was calculated according to the Reed–Muench method [27].

### Preparation of inactivated vaccines and immunization of pigs and cattle

The mutant viruses or the wt virus were collected from infected BHK-21 monolayers and inactivated with BEI. The inactivated antigens were then purified by sucrose density gradient as above described. The quantity and concentration of the 146S antigen were determined by 260 nm spectrophotometer analysis. Three water-in-oil-in-water vaccines were prepared with three inactivated viral antigens as previously described [28]. Each dose (2 mL) contained 12 µg of 146S antigen.

A total of 18 3-month-old pigs and 18 3-month-old cattle were purchased from a FMDV-seronegative farm. Pigs and cattle were housed in the animal isolation Bio-Safety Level 3 facility at the LVRI. Six pigs and six cattle were vaccinated intramuscularly in the neck with the rHN, rHN/TURVP1 or rHN/NXVP1 inactivated vaccine. Each animal received 12 µg of 146S viral antigen. All animals were given a booster inoculation at 28 dpv. On 0, 14, 21, 28, 35, 42, 49 and 56 dpv, blood samples of all animals were collected to measure the presence of anti-FMDV antibodies and virus-neutralizing antibodies.

### Liquid-phase blocking ELISA to detect anti-structural protein antibodies

Anti-FMDV structural protein antibodies in pigs and cattle vaccinated with 3 experimental vaccines were detected using a liquid-phase blocking ELISA (LPBE) as previously described [29]. Serum titres > 2.1 log_10_ were considered positive.

### Virus neutralization test (VNT) and vaccine matching

Blood samples collected from all pigs and cattle at 28 and 56 dpv were used to determine neutralizing antibody titres against the circulating FMDVs. FMDV strains O/Tibet/99, O/XJ/CHA/2017, O/NXYCh/CHA/2018 and O/GXCX/CHA/2018 belonging to four lineages of serotype O were used for the VNT. Briefly, heat-inactivated sera were twofold serially diluted (1:4, 1:8, 1:16, 1:32, 1:64, 1:128, 1:256, 1:512, 1:1024, 1:2048) with MEM in 96-well cell culture plates in a total volume of 50 µL. Then, 50 µL of an FMDV suspension containing 100 TCID_50_ was added to each well. The plate was gently agitated and incubated for 1 h at 37 °C. After incubation, 100 µL of BHK-21 cells (5.0 × 10^5^ cells/mL) were added to each well and incubated for another 48 h at 37 °C with 5% CO_2_. The wells were examined for the appearance of a CPE, and the neutralizing antibody titres were calculated as the log_10_ of the reciprocal antibody dilution required for 50% neutralization of 100 TCID_50_ of virus. All tests were repeated at least twice.

Vaccine matching was analysed using the neutralizing antibody titres of 28 primary vaccination sera collected from six cows according to the OIE Terrestrial Manual 2021 [30]. “r_1_” = the reciprocal arithmetic titre of reference serum against the field virus/the reciprocal arithmetic titre of reference serum against the vaccine virus. r_1_ values > 0.3 suggest that the field isolate is sufficiently similar to the vaccine strain and that the use of the vaccine is likely to confer protection against challenge with the field isolate. r_1_ values < 0.3 indicate that there is a significant antigenic difference between vaccine strains and field viruses, and in that case, vaccines with normal potency would probably not protect against challenge.

### Genetic analysis of FMDV variations

Two recombinant viruses were generated by substitution of the VP1 gene of O/TUR/5/2009 and O/NXYCh/CHA/2018 based on FMD vaccine strain O/HN/CHA/93. Therefore, the nucleotide and deduced amino acid sequences of VP1 genes of these viruses were aligned using DNASTAR software package version 7.0. The VP1 sequences of these viruses were obtained from GenBank.

### Ethics statement

All animal experiments were approved and performed according to the guidelines of the Gansu Ethical Review Committee (licence SYXK-GAN-2014-003).

### Statistical analysis

The statistical relationships between the recombinant vaccine groups and the control group were determined. The t tests were carried out using GraphPad Prism Software (version 5.0, GraphPad Software).

## Results

### Generation and characterization of the mutant FMDVs

**Figure 2:**
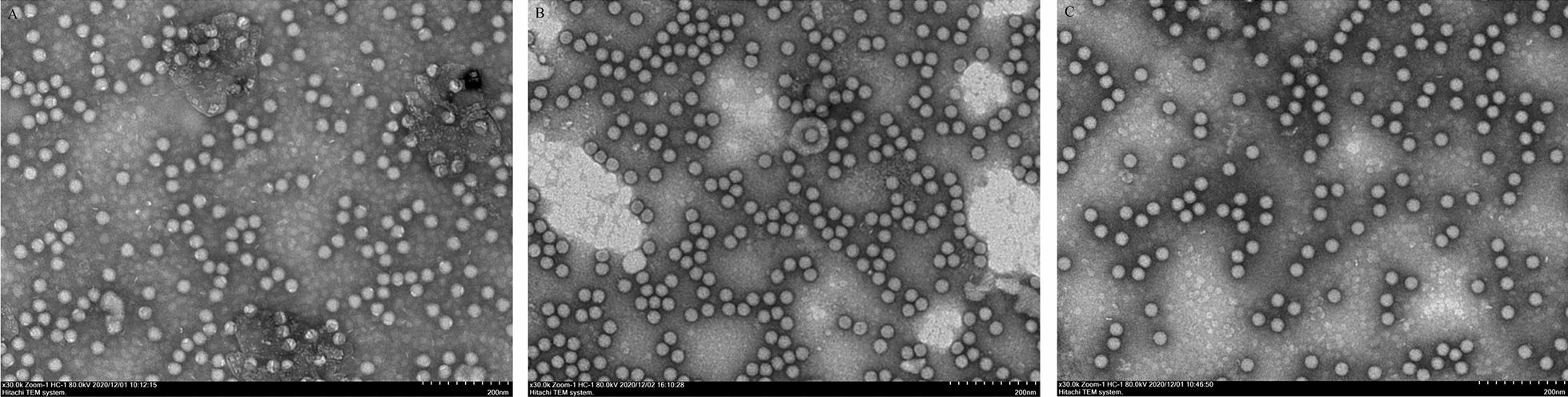
**Growth characteristics of FMDVs in BHK-21 cells.** **A** Plaque morphologies of the mutant viruses and wt virus. Monolayers were infected with tenfold serial dilutions of rHN/TURVP1, rHN/TURVP1 and rHN. After 1 h of adsorption, infected cells were overlaid with 0.6% gum tragacanth. The cells were fixed, stained and photographed at 48 h post-infection. **B** One-step growth curves for the mutant viruses and the wt virus. Monolayers were infected with rHN/TURVP1, rHN/TURVP1 and rHN (5 moi), and samples were analysed at 4, 8, 12, 16 and 20 hpi. The results were obtained from three individual experiments.

The full-length plasmids pOFS/TURVP1 and pOFS/NXVP1 containing the substitution of the VP1 genes of O/TUR/5/2009 and O/NXYCh/CHA/2018 were generated based on an infectious cDNA clone of FMDV O/HN/CHA/93 using routine molecular biological methods. The linearized constructs were transfected into BSR/T7 cells, and a CPE was readily observed 3 days post-transfection. Two viable viruses were successfully recovered from the transfected cells. To confirm that the resulting viruses contained the expected genetic substitutions, RNAs extracted from each supernatant of transfected cell cultures were amplified by RT-PCR and sequenced. The results revealed that the recombinant viruses had targeted changes. Additionally, electron microscopy also revealed that the recombinant viral particles had icosahedral symmetry, had a spherical shape, and were approximately 25–30 nm in diameter (Figure 2).

**Figure 3:**
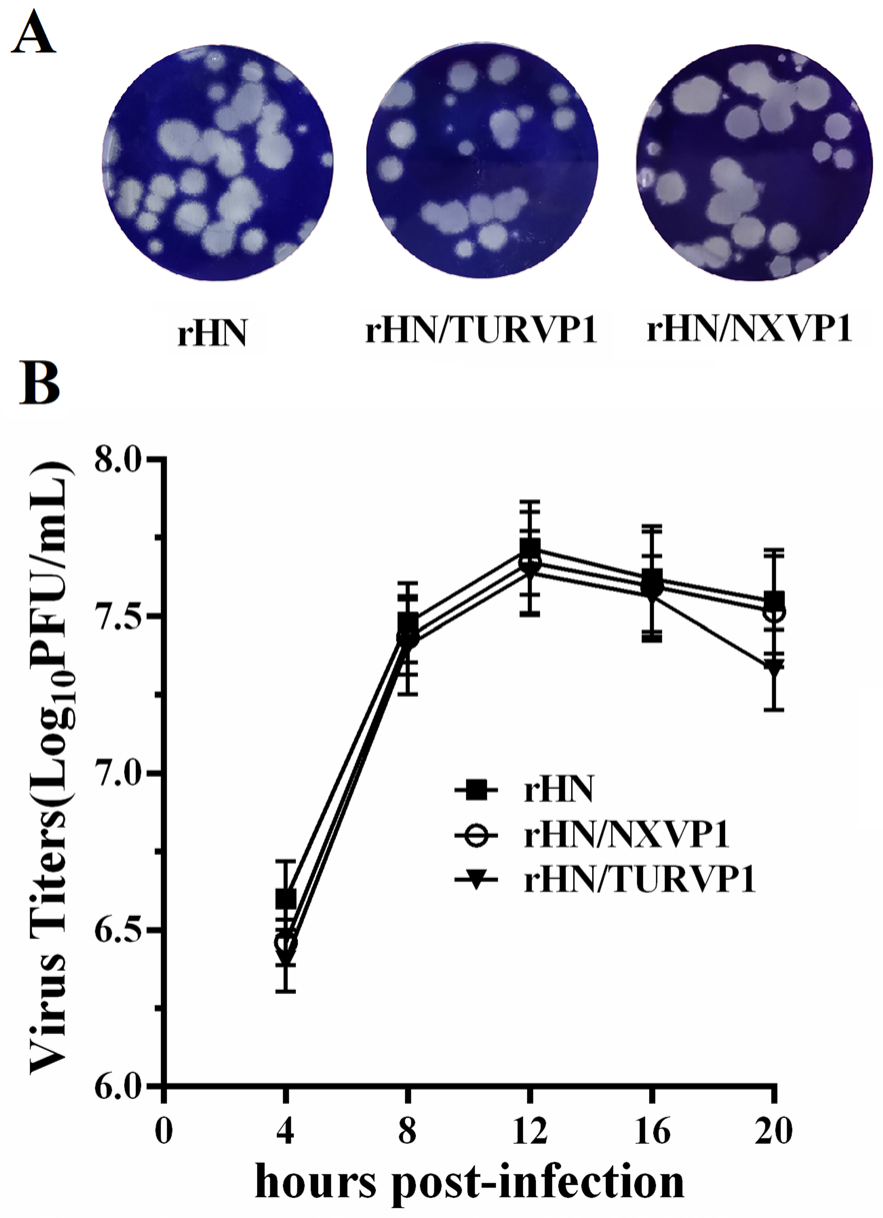
**Electron microscopy of FMDVs.** The mutant viruses and the wt virus (fifth passage) were cultured in adherent BHK-21 cells and harvested after the occurrence of 100% CPE. The virus particles were isolated by sucrose density gradient centrifugation and observed by negative-stain electron microscopy. **A** rHN/NXVP1, **B** rHN/TURVP1, **C** rHN. Bar = 100 nm.

To determine whether the substitutions of the VP1 capsid protein have some effects on the plaque phenotypes and growth characteristics in BHK-21 cell culture, plaque assays and in vitro characterization studies were performed. The results showed that the wt virus and the mutant viruses all developed visible plaques in BHK-21 cells, and the plaque phenotypes were a mix of various sizes. rHN and rHN/NXVP1 formed small (1–2 mm), medium (3–4 mm) and large (5–6 mm) plaques on BHK-21 cells, respectively. rHN/TURVP1 formed small (0.5–1 mm), medium (2–3 mm) and large (4–5 mm) plaques on BHK-21 cells (Figure 3A). In vitro growth kinetics indicated that there was no significant difference among these viruses, although the overall yields of rHN/TURVP1 were slightly lower than those of the wt virus and rHN/NXVP1 at 20 hpi (Figure 3B).

### Pathogenicity of the chimeric viruses in suckling mice

**Figure 4:**
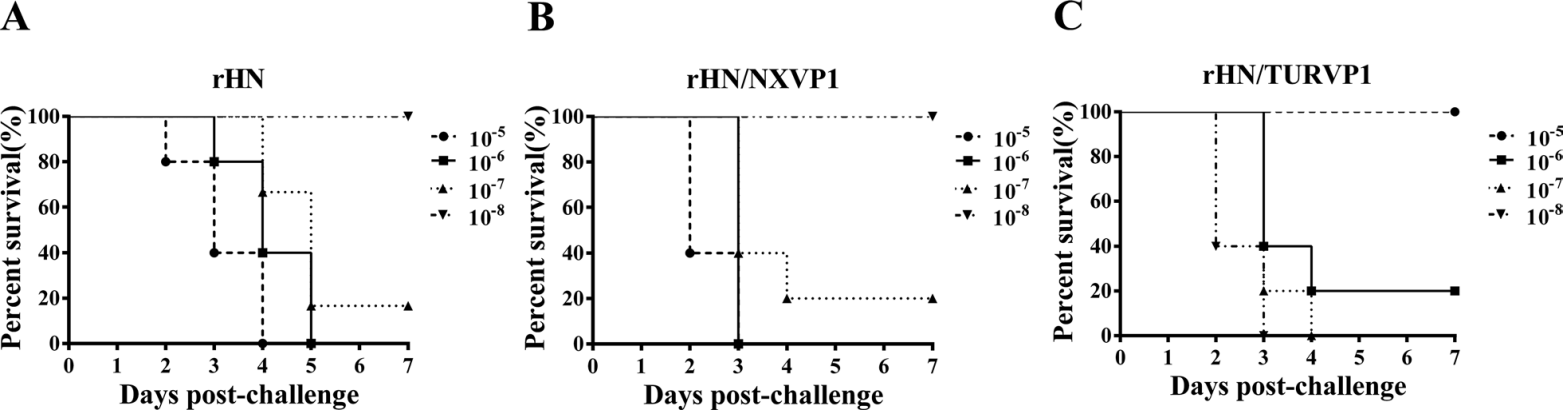
**The survival of suckling mice inoculated with FMDVs.** Two-day-old BALB/c suckling mice were inoculated IP with tenfold dilutions of the mutant viruses and the wt virus and observed for 7 days. **A** The survival rate of suckling mice inoculated with rHN. **B** The survival rate of suckling mice inoculated with rHN/NXVP1. **C** The survival rate of suckling mice inoculated with rHN/TURVP1. (black filled circle) Dilution of 10^–5^, (black filled square) dilution of 10^–6^, (black filled triangle) dilution of 10^–7^, (black filled inverted triangle) dilution of 10^–8^.

Two-day-old BALB/C suckling mice were inoculated with 0.2 mL of diluted viruses (10^–4^–10^–8^) to investigate the pathogenicity of the chimeric viruses. All suckling mice inoculated with the wt virus died within 2 to 5 days, and the suckling mice inoculated with rHN/NXVP1 and rHN/TURVP1 died within 2 to 4 days. All three viruses had an LD_50_ of 10^7.4^ TCID_50_ (Figure 4), and no difference in virulence was observed between the wt virus and the mutant viruses.

### FMDV-specific antibody responses induced by the experimental vaccines in pigs and cattle

**Figure 5:**
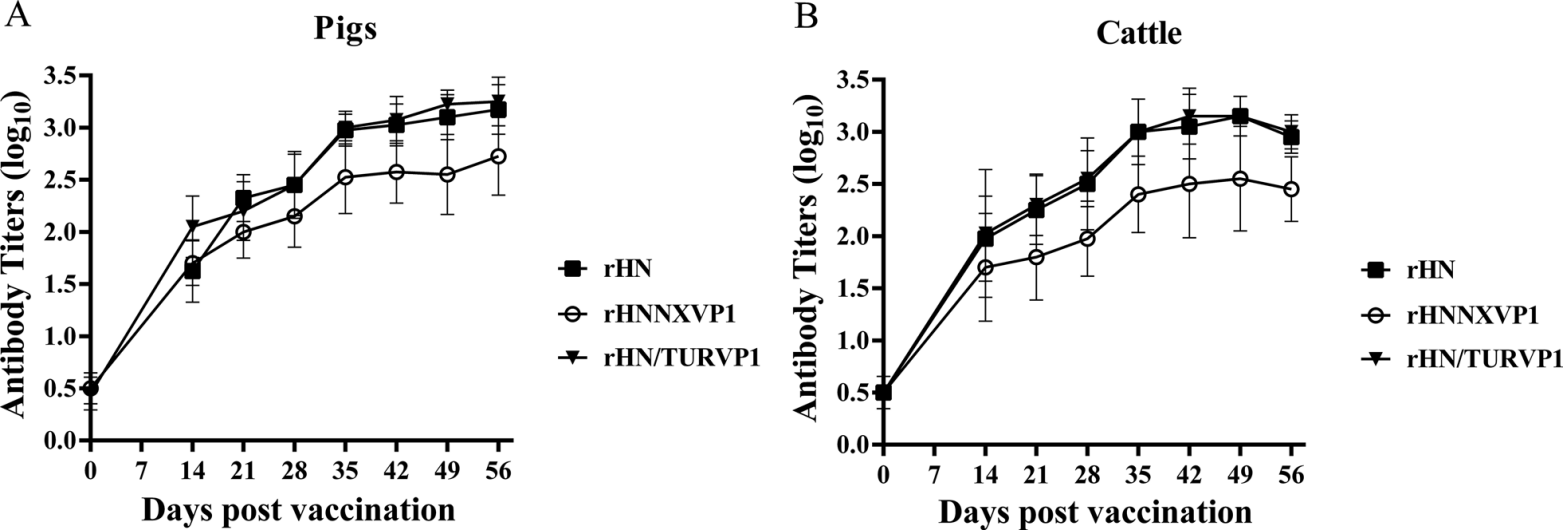
**The anti-structural protein antibodies in vaccinated pigs and cows.** Groups of six pigs and six cows were vaccinated with the mutant virus (rHN/TURVP1 or rHN/NXVP1) or the wt virus vaccine at Days 0 and 28; blood samples were assayed by liquid-phase blocking ELISA (LPBE) on Days 0, 14, 21, 28, 35, 42, 49 and 56 post-vaccination. The results were obtained from three replicates of six animals. Error bars indicate SDs from the mean.

To investigate the potential of the chimeric FMDVs as vaccine candidates, six pigs and six cattle each were inoculated with 12 μg of 146S viral antigen. The antibody response elicited in each pig and cow at 2–8 weeks post-vaccination (pv) was measured using a liquid-phase blocking ELISA (LPBE) kit. The results showed that the anti-structural protein antibodies in all pig and cattle sera were substantially enriched after vaccination. Antibody titres ≥ 1.65 log_10_ were observed in all pigs vaccinated with the rHN/NXVP1 vaccine, and the corresponding mean antibody titre of the 6 pigs was >2.1 log_10_ at 28 dpv (Figure 5A). Antibody titres >1.95 log_10_ were observed in all pigs vaccinated with the rHN/TURVP1 and wt virus vaccines, and the corresponding mean antibody titres of the 6 pigs were > 2.4 log_10_ at 28 dpv (Figure 5A). Antibody titres ≥ 1.65 log_10_ were observed in all cattle vaccinated with the rHN/NXVP1 and wt virus vaccines, and the corresponding mean antibody titre of 6 cattle vaccinated with rHN/NXVP1 was > 1.8 log_10_, whereas that of those vaccinated with rHN was >2.1 log_10_ at 28 dpv (Figure 5B). Antibody titres ≥2.1 log_10_ were observed in all cattle vaccinated with rHN/TURVP1, and the corresponding mean antibody titre of the 6 cattle was >2.4 log_10_ at 28 dpv (Figure 5B). After boosting vaccination at 28 dpv, the antibody levels in pig and cattle sera were further increased, the mean antibody titres of 6 pigs reached the highest level at 56 dpv, and that of 6 cattle reached the highest level at 49 dpv (Figures 5A, B). The highest mean antibody titre of the 6 pigs vaccinated with rHN/NXVP1 was >2.7 log_10_, and that of those vaccinated with rHN/TURVP1 or rHN was > 3.0 log_10_ (Figure 5A). The highest mean antibody titre of 6 cattle vaccinated with rHN/NXVP1 was >2.6 log_10_, and that of those vaccinated with rHN/TURVP1 or rHN was > 3.0 log_10_ (Figure 5B). These results indicated that the mean anti-structural protein antibody titres of the sera collected from animals vaccinated with the wt virus and rHN/TURVP1 vaccines were obviously higher than those of the animals vaccinated with the rHN/NXVP1 vaccine from 28 to 56 dpv (*p* < 0.05), indicating that the animals vaccinated with the wt and the rHN/TURVP1 vaccines elicited higher levels of anti-structural protein antibodies after the second vaccination compared with those elicited after the rHN/NXVP1 vaccine vaccination.

### Cross neutralization analysis in vaccinated pigs and cattle sera

**Figure 6:**
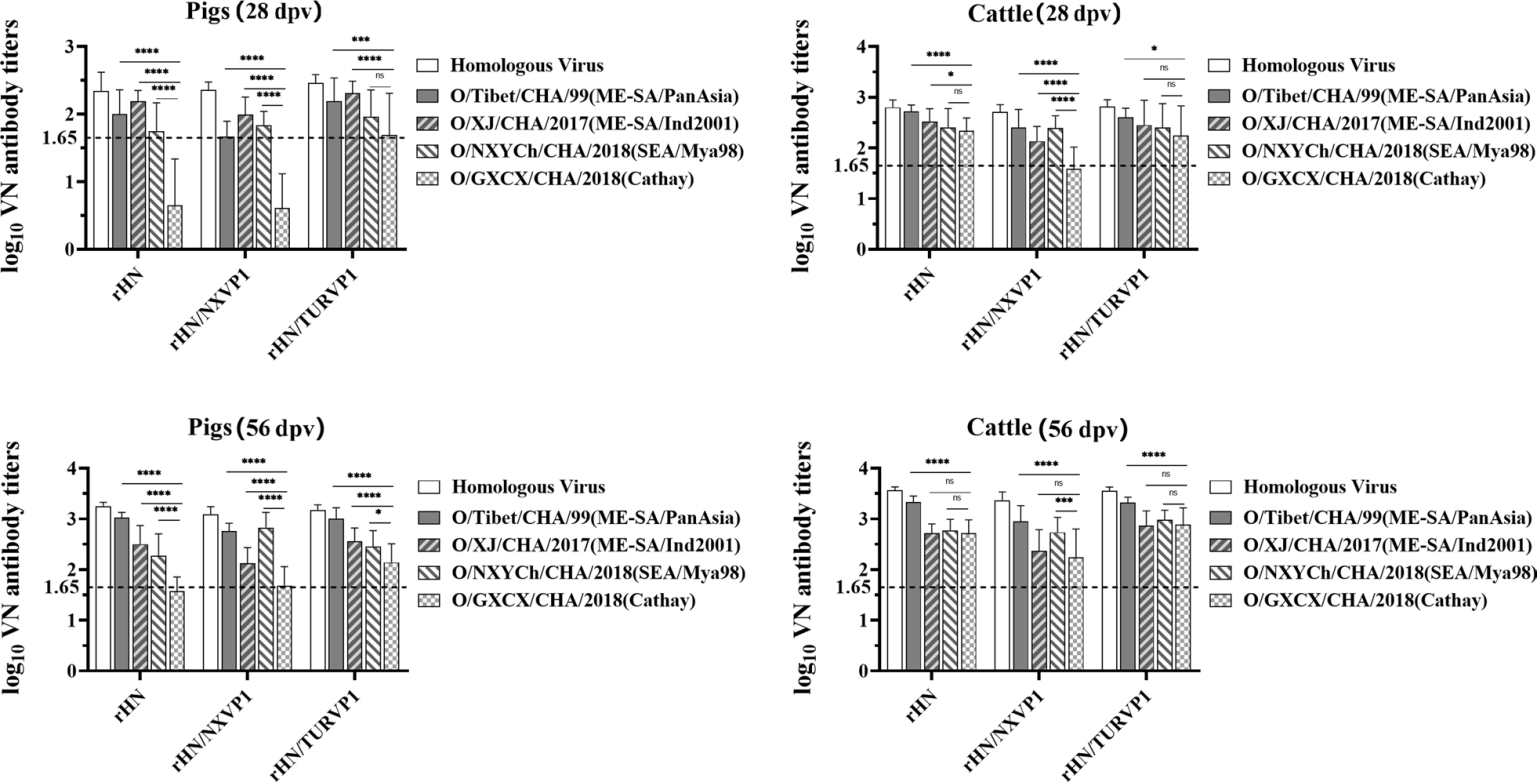
**Comparisons of the virus neutralization antibody titres against homologous and heterologous viruses in immunized pigs and cattle.** Groups of six pigs or six cattle were vaccinated with either the mutant virus (rHN/TURVP1 or rHN/NXVP1) or the wt virus vaccines on Days 0 and 28; blood samples were assayed by the virus neutralization test on Days 28 and 56 post-vaccination. The results were obtained from three replicates of six animals. Error bars indicate SDs from the mean. Statistical analysis was conducted using an unpaired t test (*p < 0.05, **p < 0.01, ***p < 0.001, ****p < 0.0001, ns: not significant). **A** Mean virus-neutralizing antibody titre (VNT (log_10_)) of six pigs vaccinated with three experimental vaccines at 28 dpv. **B** Mean VNT of six cows vaccinated with three experimental vaccines at 28 dpv. **C** Mean VNT of six pigs vaccinated with three experimental vaccines at 56 dpv. **D** Mean VNT of six cows vaccinated with three experimental vaccines at 56 dpv. The dotted line indicates the 1.65 log_10_ VNT cut-off level.

The virus-neutralizing antibodies against the heterologous viruses were evaluated using the sera collected from all pigs and cattle vaccinated with the three experimental vaccines at 28 and 56 dpv. The vaccinated pigs produced protective mean neutralizing antibody levels (>1.65 log_10_, which was considered protective [30]) against O/Tibet/99, O/XJ/CHA/2017 and O/NXYCh/CHA/2018, while they all induced relatively low mean neutralizing antibody titres to O/GXCX/CHA/2018 at 28 dpv; however, only the pigs vaccinated with the rHN/TURVP1 vaccine produced protective mean neutralizing antibodies against O/GXCX/CHA/2018 when compared to that of rHN and rHN/NXVP1 at 28 dpv (Figure 6A). The mean VN titres against four heterologous viruses in all pigs further increased at 56 dpv, and only the pigs vaccinated with the rHN/TURVP1 vaccine produced high mean VN titres (>2.1 log_10_) against O/GXCX/CHA/2018 at 56 dpv (Figure 6C). In cattle, the mean VN titres against four heterologous viruses were ≥2.1 log_10_ in the wt virus and the rHN/TURVP1 vaccine groups, the mean VN titres against O/Tibet/99, O/XJ/CHA/2017, and O/NXYCh/CHA/2018 were ≥2.1 log_10_ and against O/GXCX/CHA/2018 were <log_10_ 1.65 in the rHN/NXVP1 vaccine group at 28 dpv (Figure 6B). The mean VN titres against four heterologous viruses were ≥2.7 log_10_ in the wt and rHN/TURVP1 vaccine groups and ≥2.1 log_10_ in the rHN/NXVP1 vaccine group at 56 dpv (Figure 6D). These results indicated that the rHN/TURVP1 vaccine showed relatively better broad-range cross-reactivity against the field isolates of four lineages of serotype O FMDV in pigs and cattle than the wt virus and the rHN/NXVP1 vaccines.

### Vaccine matching using vaccinated sera

**Figure 7:**
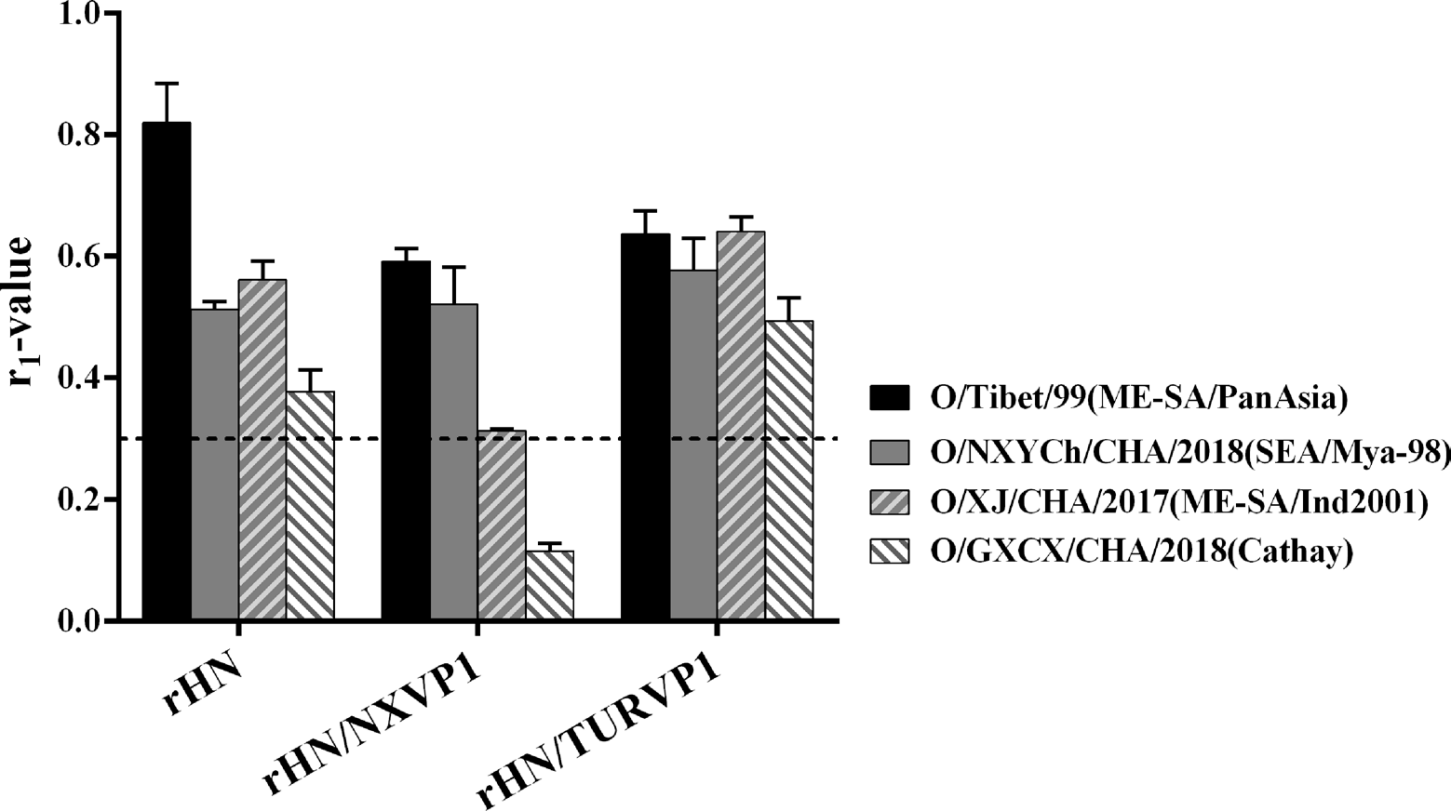
**Serological cross-reactive responses against heterologous FMDVs.** A two-dimensional (2D) virus neutralization test was performed using sera from six cows vaccinated with the rHN, rHN/NXVP1 or rHN/TURVP1 vaccine at 28 dpv. The dotted line indicates the cut-off value of 0.3. Three experiments were performed independently, and the mean r_1_ values were obtained from three replicates of six animals. Error bars indicate SDs from the mean.

The vaccine matching relationship (r_1_) values were calculated by 2D VNT using serum samples collected from six cows inoculated with the experimental vaccines at 28 dpv. The r_1_ value between the wt virus and heterologous viruses (O/Tibet/99, O/XJ/CHA/2017, O/NXYCh/CHA/2018 and O/GXCX/CHA/2018) as well as the rHN/TURVP1 and four heterologous viruses was > 0.3 (Figure 7), that between rHN/NXVP1 and O/Tibet/99, O/XJ/CHA/2017, and O/NXYCh/CHA/2018 was > 0.3, while that for O/GXCX/CHA/2018 was < 0.3 (Figure 7), indicating that the field isolates are sufficiently similar to the rHN/TURVP1 and the wt virus and that there is a significant antigenic difference between the field isolates and rHN/NXVP1.

### Genetic analysis of FMDVs

**Figure 8:**
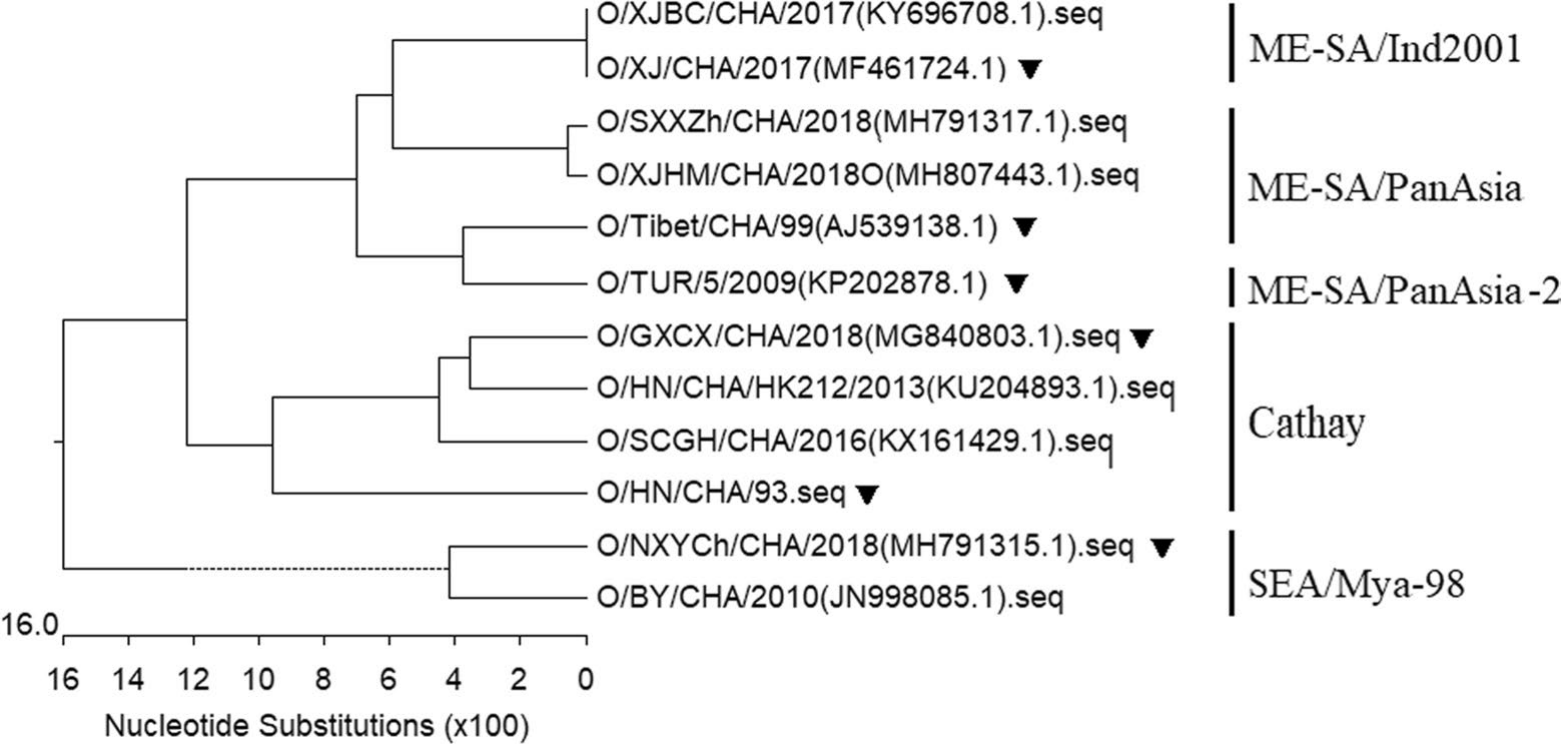
**Phylogenetic tree of FMDVs.** A phylogenetic tree based on the VP1 nucleotide sequences was produced using MEGA 7.0. The VP1 sequences of FMDVs were obtained from GenBank. The viruses used in this study are marked with an inverted triangle (▼).

**Figure 9:**
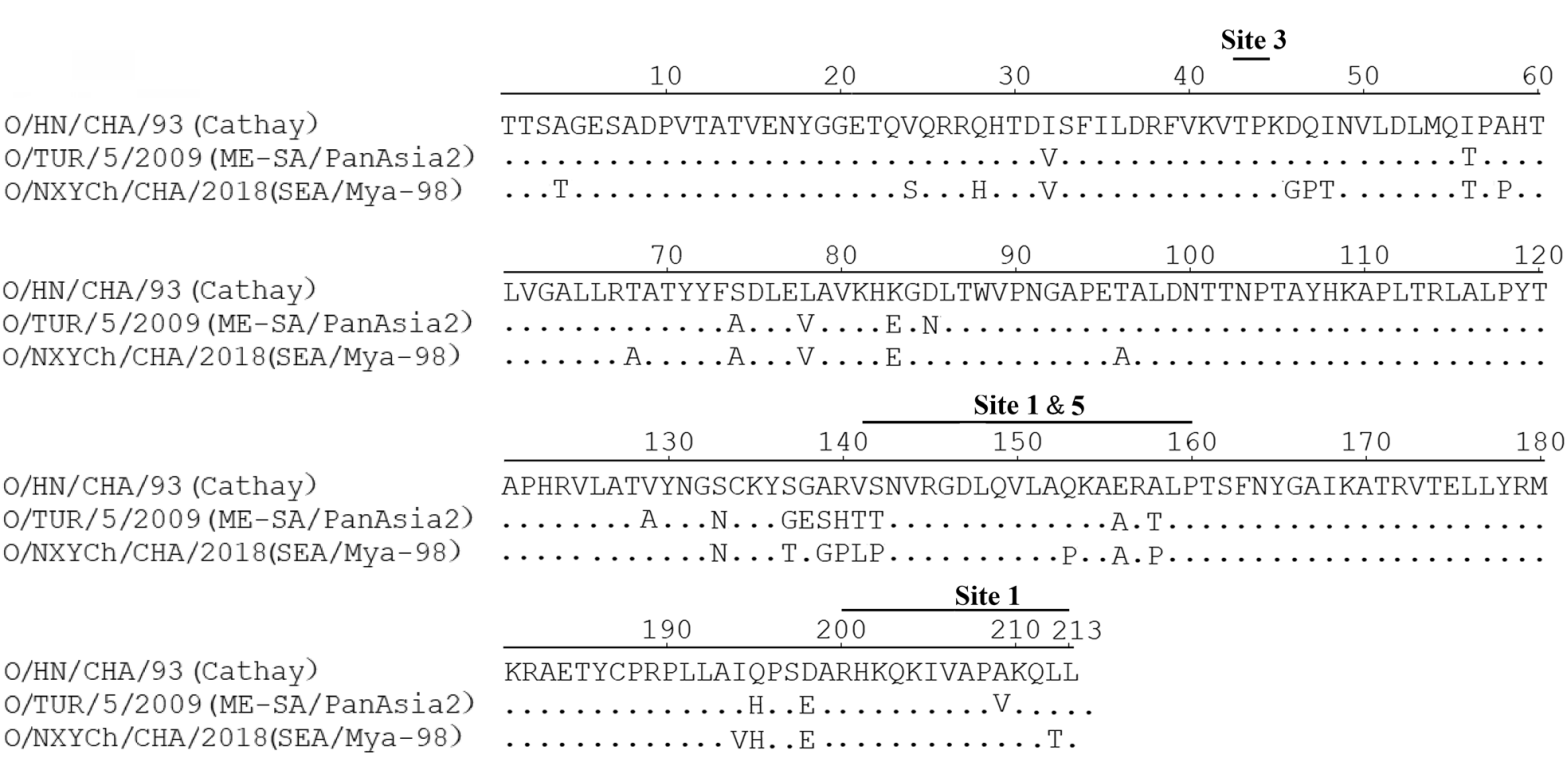
**Comparison of amino acid sequences of the VP1 protein of FMDVs.** Amino acid sequences of VP1 of O/HN/CHA/93, O/TUR/5/2009 and O/NXYCh/CHA/2018 were aligned using DNASTAR software package version 7.0. The VP1 sequences of these viruses were obtained from GenBank.

The analysis of the VP1 nucleotide sequences of O/HN/CHA/93, O/TUR/5/2009 and O/NXYCh/CHA/2018 showed that there was 82.6% nucleotide identity between O/HN/CHA/93 and O/TUR/5/2009 and 78.9% nucleotide identity between O/HN/CHA/93 and O/NXYCh/CHA/2018 (data not shown). O/HN/CHA/93, O/TUR/5/2009 and O/NXYCh/CHA/2018 belong to the Cathay, ME-SA and SEA topotypes, respectively (Figure 8). The alignment of deduced amino acid sequences of the VP1 gene of O/HN/CHA/93, O/TUR/5/2009 and O/NXYCh/CHA/2018 revealed that O/TUR/5/2009 showed variation at 18 amino acid positions and that of O/NXYCh/CHA/2018 appeared to vary at 25 amino acid positions compared with that of O/HN/CHA/93 (Figure 9). There are 20 amino acid changes between O/TUR/5/2009 and O/NXYCh/CHA/2018. Some changes appeared in antigenic sites, while some did not (Figure 9). No change existed at the key residues (residues 43, 44, 144, 148, 149, 150 and 208) of the three antigenic sites of VP1, and many mutations occurred in the G-H loop spanning residues 133–160 (Figure 9).

## Discussion

In recent years, serotype O FMDV has become widely prevalent in China and has evolved into four distinct lineages named O/SEA/Mya-98, O/ME-SA/PanAsia, O/ME-SA/Ind-2001 and O/Cathay [31]. The ME-SA topotype is divided into three lineages designated PanAsia, PanAsia-2 and Ind2001 [32]. Among them, the PanAsia lineage has been popular for many years in China [31], and the transboundary incursions of Ind2001 lineages were reported in 2017 [31], while the PanAsia-2 lineage is prevalent in neighbouring countries and caused recent incursions into other countries [32], but it has not been detected in China until now. Therefore, the current complex epidemiological situation and the risk of transboundary incursions of PanAsia-2 lineages emphasize the importance of screening for a matched vaccine strain with broad antigenic coverage for type O FMDV in China.

Reverse genetic technology provides a powerful tool for the development of potential FMD vaccines by replacing the whole (P1) or partial capsid gene (VP231 or VP1) of appropriate field isolates [33–36]. Here, we generated two chimeric FMDVs featuring the substitution of the VP1 gene of the O/TUR/5/2009 vaccine strain and the O/NXYCh/CHA/2018 epidemic strain by reverse genetics. Evaluation of the plaque phenotypes of the chimeric viruses showed that the plaque sizes of rHN and rHN/NXVP1 were slightly larger than those of rHN/TURVP1, which can probably be attributed to the differences in amino acids in the VP1 protein. One-step growth kinetics revealed that the mutant viruses grew similarly to the wt virus, although the peak titres of rHN/TURVP1 at 20 h post-infection were slightly lower than those of rHN and rHN/NXVP1; a lower rate of virus particle release from infected cells at this time may cause this difference [37].

A highly immunogenic vaccine is pivotal for protection against FMDV [38]. In the present study, the mean anti-structural protein antibody levels of the pigs and cows vaccinated with the rHN/TURVP1 and rHN vaccines were higher than those of the pigs vaccinated with the rHN/NXVP1 vaccine at 28–56 dpv, indicating that the replacement with the different VP1 proteins has a different effect on the immunological responses in animals, which may be attributed to the differences in the amino acids of the VP1 protein. The additional vaccination with the three vaccines induced the production of high levels of antibodies, indicating the effectiveness of the second injection.

FMD vaccine candidates with a good match with field isolates are very important for the successful implementation of FMD control programmes based on routine vaccination [8]. It was reported that there are two different methods to screen for the most suitable vaccine to combat FMD: in vivo and in vitro approaches [23]. However, in vivo testing is labour intensive, time-consuming, and costly and requires high-containment biosecurity facilities; thus far, few studies have reported attempts to observe cross-protection directly [23]. It is generally proposed that VNT is a widely used approach for preliminary selection of the most appropriate vaccine strains for protection against field isolates [8, 39]. In this study, we used VNT to analyse the capacity of cross-reactive responses of three experimental vaccines against field isolates of four lineages of serotype O FMDV. The rHN/TURVP1 vaccine could induce the production of protective neutralizing antibodies against the circulating FMDVs of four lineages in pigs and cattle at 28 dpv, while the wt and rHN/NXVP1 vaccines did not, indicating that only the rHN/TURVP1 vaccine showed relatively good broad-range cross-reactivity against the field isolates of four lineages. In particular, rHN and O/GXCX/CHA/2018 both belong to the Cathay topotype, and six pigs vaccinated with this vaccine produced very low VN antibodies or did not produce VN antibodies against O/GXCX/CHA/2018 at 28 dpv. The analysis of the VP1 sequences of O/HN/CHA/93 and O/GXCX/CHA/2018 showed approximately 83.9% nucleotide identity and 88.7% amino acid similarity (data not shown), indicating that the circulating field isolate of the Cathay topotype exhibits high levels of genetic variation. Additionally, the vaccine matching relationship (r_1_) analysis showed that only the r_1_ value between the rHN/NXVP1 and O/GXCX/CHA/2018 was <0.3, demonstrating that there is a significant antigenic difference between rHN/NXVP1 and O/GXCX/CHA/2018, while the wt virus and rHN/TURVP1 were both similar to the field isolates belonging to four lineages of serotype O. Although the virus tested generated an r_1_ value greater than 0.3, which is considered indicative of a good antigenic match, many studies have shown that high-potency FMDV vaccines may still provide protection against field viruses even when the r_1_ values are less than 0.3 [40–42]. In this study, we used the high-potency vaccine (12 µg/dose); however, 5/6 pigs vaccinated with the wt virus and rHN/NXVP1 vaccine produced very low levels of (<1.34 log_10_) or did not produce neutralizing antibodies against the circulating virus of the Cathay topotype at 28 dpv. In general, in vitro neutralization titres are closely correlated with protection in vivo, although the animals with low VN titres and without measurable VN titres were protective [40]. Overall, we suggest that the existing vaccine strain (O/HN/CHA/93) is less suitable for use as a vaccine to provide protection against serotype O FMDVs circulating in China, especially with the emergence of a new variant of the Cathay topotype, while the recombinant virus containing the VP1 capsid protein of the O/TUR/5/2009 vaccine strain has potential for the development of an emergency vaccine with a broad antigenic spectrum for the prevention and control of the circulating serotype O FMDVs in China, although it is necessary to further measure cross-protection using an in vivo matching test. The difference in serological cross-reactivity of these vaccines may be attributed to the changes in some noncritical residues located at the antigenic site of the VP1 protein.

In summary, our studies provide vital information to help understand whether chimeric viruses have the potential to be used as vaccine candidates for protection against circulating serotype O FMDVs in China and can provide a theoretical basis for in vivo challenge tests in the future.
